# Fatal Nonvariceal Gastrointestinal Bleeding in a Cirrhotic Patient Taking Apixaban with No History of Hemorrhage

**DOI:** 10.7759/cureus.6126

**Published:** 2019-11-11

**Authors:** Hung-Wen Yen

**Affiliations:** 1 Internal Medicine, Louise A. Weiss Memorial Hospital, Chicago, USA

**Keywords:** cirrhosis, gi bleeding, anticoagulation, doac

## Abstract

This report describes a cirrhotic female patient with no history of bleeding or other gastrointestinal disorder who experienced fatal gastrointestinal bleeding (GIB) after taking apixaban [which is a direct oral anticoagulant (DOAC)] for one month for management of chronic nonvalvular atrial fibrillation. The use of apixaban, coupled with the predilection for hemorrhage in cirrhotic patients, most likely contributed to her fatal GIB. Caution should be exercised in considering apixaban and potentially other members of the DOAC class for the treatment of cirrhotic patients until further research can explore the safety of DOAC therapy in cirrhotic patients.

## Introduction

Individuals with chronic liver disease, especially those in end-stage liver failure, have an increased risk of clinical bleeding as well as thrombosis [1]. The bleeding diathesis of cirrhosis is associated with abnormalities in all stages of the hemostatic process, including platelet dysfunction, coagulopathy, and abnormal fibrinolysis [2]. Variceal hemorrhage is commonly found in 25%-35% of patients with cirrhosis and accounts for 80%-90% of bleeding episodes in these patients [3]. However, nonvariceal gastrointestinal bleeding (GIB) is much less common in patients with cirrhosis; one study reported nonvariceal GIB occurred in only 13% of inpatient admissions for decompensated cirrhosis, and all reported nonvariceal GIB cases were secondary to other concomitant gastrointestinal diseases [4].

Despite the increased bleeding risk for cirrhotic patients, major bleeding is uncommon in cirrhotic patients on direct oral anticoagulant (DOAC) therapy [5]. This report describes a cirrhotic female patient with no history of bleeding or other gastrointestinal disorder who experienced fatal GIB after taking apixaban for one month.

## Case presentation

A 64-year-old woman presented to the ED with mild generalized upper abdominal pain, hematemesis, and melena that persisted for three days prior to admission. Her previous medical history included type 2 diabetes, hypertension, Child-Turcotte-Pugh (CTP) class B liver cirrhosis secondary to nonalcoholic steatohepatitis for 13 years, congestive heart failure with preserved ejection fraction, and paroxysmal atrial fibrillation (Afib). One month prior to presentation, her treatment for Afib was switched from oral aspirin 81 mg once daily to oral apixaban 5 mg twice daily; her apixaban had run out three days prior to admission. She was also taking 200 mg/day oral amiodarone. Her initial vital signs were stable, with a blood pressure of 110/53 mmHg. Physical examination showed that her abdomen was mildly distended, with tenderness over the epigastric region and right upper quadrant. Laboratory data on admission included a hemoglobin concentration of 9.2 g/dL, which had decreased from 11.1 g/dL documented five months earlier. Her leukocyte count was 10.2 x 103/µL (reference range: 4.8-10.8 x 103/µL) , her platelet count was 137 x 103/µL (reference range: 150-450 x 103/µL), her INR was 1.5, and her partial thromboplastin time (PTT) was 29.1 s. She had a blood urea nitrogen (BUN) concentration of 47 mg/dL, a serum creatinine concentration of 0.95 mg/dL, a serum albumin concentration of 2.1 g/dL, an alanine aminotransferase concentration of 62 IU/L, an aspartate aminotransferase concentration of 148 IU/L, and a total bilirubin concentration of 1.5 mg /dL. Other laboratory findings were unremarkable.

Five hours after arrival at the ED, the patient was admitted to the general medical ward and started on a pantoprazole drip. Seven hours after ED arrival, she experienced roughly 500 mL of coffee-ground emesis. Repeat laboratory examination showed a hemoglobin concentration of 8.1 g/dL, a BUN concentration of 47 mg/dL, a serum creatinine concentration of 1.08 mg/dL, an INR of 1.7, and a PTT of 33 s. The patient was transferred to the intensive care unit (ICU) for management of a suspected active GIB.

After ICU transfer, the patient received four units of packed red blood cells, three units of fresh frozen plasma, and one unit of plateletpheresis. An emergency esophagogastroduodenoscopy (EGD) showed a significant quantity of blood pooling along the lesser curvature, likely from an arterial source. The EGD was unable to directly visualize the bleeding source, as the bleeding did not stop. The EGD showed a normal esophagus without varices and a normal antrum, pylorus, and duodenal bulb with no evidence of bleeding. Immediate transfer to a tertiary medical center was recommended, as angioembolization could not be performed successfully at our community hospital. However, her blood pressure continued to decrease rapidly despite being on multiple vasopressors. Severe lactate acidosis, with serum lactate acid levels peaking at 19.3 mmol/L, resulted in pulseless electrical activity while prothrombin complex concentrate (PCC) was being prepared. Advanced cardiac life support was initiated, but the efforts were unsuccessful, and the patient died 15 hours after ED arrival.

## Discussion

To our knowledge, this is the first reported case of fatal GIB in a patient with liver cirrhosis taking apixaban in the English literature. Two review articles published in 2019 confirmed no reported fatal GIB in patients with liver cirrhosis taking apixaban to date [5-6]. Although major bleeding, including intracranial hemorrhage and GIB, has been reported in several cirrhotic patients on chronic DOAC, none were fatal [5, 7]. The use of DOACs in cirrhotic patients remains uncommon, as their safety has not been established in these patients [8-9]. Although most clinical trials of DOACs have excluded patients with impaired liver function [9], several studies have reported that the efficacy and safety of DOACs in cirrhotic patients were noninferior if not superior to traditional vitamin K antagonists (VKA), with similar or even lower risks of GIB and major bleeding [5, 7-9]. In addition to being less common, major bleeding was found to occur later in patients on DOACs than on VKAs, with peak incidence after one year on DOACs, but within the first 30 days on VKAs [7]. Subgroup analyses of a retrospective cohort study found that the risk of major bleeding was lower in cirrhotic patients taking dabigatran or rivaroxaban, but not apixaban, than in patients taking warfarin [5], in good agreement with the relative proportions of DOACs eliminated through the liver, which were shown to be 75% for apixaban, 65% for rivaroxaban, 50% for edoxaban, and 20% for dabigatran [5-6]. The US Food and Drug Administration (FDA) guidelines recommend that DOAC dosage in individuals with liver disease be based on CTP class, with all DOACs being indicated for and requiring no dose adjustment in patients with CTP class A cirrhosis [6, 9-12]. Extra caution is recommended for patients with CTP class B cirrhosis, whereas DOACs are not recommended for patients with CTP class C cirrhosis. To date, there are no specific recommendations for dabigatran and apixaban treatment of patients with CTP class B cirrhosis, although a single 150-mg dose of dabigatran provides similar drug exposure and coagulation indices in healthy individuals and patients with CTP class B cirrhosis [9-10, 13].

In contrast, rivaroxaban and edoxaban are not recommended for patients with CTP class B or C liver cirrhosis [11-12]. A meta-analysis found no association between DOACs and significant risks of drug-induced liver injury [14]. Moreover, the US FDA has approved the use of andexanet for the reversal of anticoagulation induced by rivaroxaban or apixaban in patients with life-threatening and uncontrolled bleeding [6, 15].

The patient described in this report had no history of bleeding or other gastrointestinal condition other than liver cirrhosis prior to admission. Moreover, EGD showed that her esophagus, antrum, pylorus, and duodenal bulb were normal. The combination of apixaban treatment and the hemostatic abnormalities associated with liver cirrhosis, therefore, may have contributed to her fatal GIB. She had been exposed to apixaban for only one month prior to her fatal upper GIB, a much shorter exposure than other patients who experienced major bleeding episodes while on this drug [5, 7-8]. Her rapid deterioration following the major GIB prevented the initiation of other potential life-saving treatments, including arterial embolization, PCC, or even andexanet administration.

The outcome of the patient’s major GIB might have been more favorable if she had been on dabigatran rather than apixaban. Of all DOACs, dabigatran has the lowest proportion of hepatic elimination, which may lower the risk of toxicity of DOAC overdose, and reduce the likelihood of bleeding. In addition, idarucizumab can immediately reverse the anticoagulation of dabigatran [16]. GIB risk assessment by EGD prior to the initiation of DOAC use in patients with cirrhosis had been recommended in one review article, but no pertinent guideline has been established to date [6]. Further research on the efficacy and safety of various DOACs in cirrhotic patients may enable better selection of anticoagulant in this group of patients.

## Conclusions

This case report described a cirrhotic female patient with no history of bleeding or other gastrointestinal disorder who experienced fatal GIB following apixaban treatment for one month to manage her chronic nonvalvular Afib. This case demonstrates that fatal GIB can still occur in cirrhotic patients with no previous history of bleeding or other gastrointestinal disorder taking short-term apixaban, and physicians should exercise caution in selecting a DOAC for the treatment of cirrhotic patients. Dabigatran may be a better choice than apixaban for anticoagulation in cirrhotic patients.

## References

[1] Tripodi A, Mannucci PM (2011). The coagulopathy of chronic liver disease. N Engl J Med.

[2] Northup PG, Caldwell SH (2013). Coagulation in liver disease: a guide for the clinician. Clin Gastroenterol Hepatol.

[3] Sharara AI, Rockey DC (2001). Gastroesophageal variceal hemorrhage. N Engl J Med.

[4] Shah NL, Northup PG, Caldwell SH (2012). A clinical survey of bleeding, thrombosis, and blood product use in decompensated cirrhosis patients. Ann Hepatol.

[5] Lee HF, Chan YH, Chang SH (2019). Effectiveness and safety of non-vitamin K antagonist oral anticoagulant and warfarin in cirrhotic patients with nonvalvular atrial fibrillation. J Am Heart Assoc.

[6] Elhosseiny S, Moussawi HA, Chalhoub JM (2019). Direct oral anticoagulants in cirrhotic patients: current evidence and clinical observations. Can J Gastroenterol Hepatol.

[7] Hum J, Shatzel JJ, Jou JH, Deloughery TG (2017). The efficacy and safety of direct oral anticoagulants vs traditional anticoagulants in cirrhosis. Eur J Haematol.

[8] Intagliata NM, Maitland H, Northup PG, Caldwell SH (2015). Treating thrombosis in cirrhosis patients with new oral agents: ready or not?. Hepatology.

[9] (2015). Pradaxa (dabigatran etexilate), [package insert], Boehringer Ingelheim Pharmaceuticals Inc., Ridgefield, CA, USA. Boehringer Ingelheim Pharmaceuticals Inc., Ridgefield, CA, USA.

[10] (Initial US approval 2012, revised in June 2019). Eliquis (apixaban), [package insert], Bristol-Myers Squibb, New York, NY, USA. https://packageinserts.bms.com/pi/pi_eliquis.pdf.

[11] (2015). Xarelto (rivaroxaban), [package insert], Janssen Pharmaceuticals Inc., Titusville, NJ, USA. Janssen Pharmaceuticals Inc., Titusville, NJ, USA.

[12] (2015). Savaysa (edoxaban), [package insert], Daiichi Sankyo, Tokyo, Japan. https://www.accessdata.fda.gov/drugsatfda_docs/label/2015/206316lbl.pdf.

[13] Stangier J, Stahle H, Rathgen K, Roth W, Shakeri-Nejad K (2008). Pharmacokinetics and pharmacodynamics of dabigatran etexilate, an oral direct thrombin inhibitor, are not affected by moderate hepatic impairment. J Clin Pharmacol.

[14] Caldeira D, Barra M, Santos AT, de Abreu D, Pinto FJ, Ferreira JJ, Costa J (2014). Risk of drug-induced liver injury with the new oral anticoagulants: systematic review and meta-analysis. Heart.

[15] Siegal DM, Curnutte JT, Connolly SJ (2015). Andexanet alfa for the reversal of factor Xa inhibitor activity. N Engl J Med.

[16] Levy JH, Douketis J, Weitz JI (2018). Reversal agents for non-vitamin K antagonist oral anticoagulants. Nat Rev Cardiol.

